# An Approach to Cell Motility as a Key Mechanism in Oncology

**DOI:** 10.3390/cancers13143576

**Published:** 2021-07-16

**Authors:** José I. López, Ildefonso M. De la Fuente

**Affiliations:** 1 Department of Pathology, Cruces University Hospital, 48903 Barakaldo, Spain; 2 Biocruces-Bizkaia Health Research Institute, 48903 Barakaldo, Spain; 3 Department of Nutrition, CEBAS-CSIC Institute, Espinardo University Campus, 30100 Murcia, Spain; 4 Department of Mathematics, Faculty of Science and Technology, University of the Basque Country, 48940 Leioa, Spain

Motility is an inherent characteristic of living cells manifesting cell migration, a fundamental mechanism of survival and development. In unicellular organisms, cell migration is needed to prey and escape from predators. In multicellular individuals, however, embryogenesis, tissue repair, and adaptation to external changes do happen through cell migration. Cancer cells also display motile abilities; actually, aggressiveness in most malignant tumors depends fundamentally on two properties related to cell motility: local invasion and metastases. 

This Special Issue contains up to fourteen contributions focusing on the cell motility/cancer binomial from very different approaches and tries to serve as a showcase/sample book of the enormous possibilities still pending to be analyzed and discovered in the field. Eight articles and six reviews are in this issue. An international cast of contributors has deepened in a broad spectrum of specific processes related to cell migration in breast and colorectal cancers, as well as in rhabdomyosarcoma, melanoma, and Leydig cell tumor of the testis. The reviews revisit several basic mechanisms related to drug resistance, epithelial-to-mesenchymal transition processes, and the transfer of knowledge related to motility from single organisms to cancer cells. Finally, an ecological approach to cancer biology highlights the benefit obtained to sum on the oncology board allied scientific disciplines.

Rhabdomyosarcoma is a malignant mesenchymal neoplasm more frequently diagnosed in childhood and adolescence, where it pursues an aggressive clinical course with 3-year survival rates of only 25%. Skrzypek et al. [1] demonstrate for the first time that Snail, a transcription factor linked to E-cadherin regulation in epithelial to mesenchymal transition processes, also regulates the metastatic behavior of rhabdomyosarcoma cells, both in vivo and in vitro, promoting cell motility, invasion, and chemotaxis. This effect is accomplished by upregulating the protein expression of Ezrin and Akt. Besides, the authors have shown that the Snail-miRNA axis regulates motility of rhabdomyosarcoma cells, especially miR-28-3p through indirect modulation of Ezrin levels. The authors conclude that this new regulatory mechanism of cell motility in this type of sarcoma could be shared by other mesenchymal neoplasms and propose to consider Snail a potential new target in future therapy modalities.

Colorectal adenocarcinoma is a common neoplasm in Western countries and a paradigmatic example of intratumor heterogeneity. Kryczka et al. [2] have observed that ABCC4, a protein belonging to a superfamily of ATP-binding cassette proteins, can regulate cell migration in colorectal adenocarcinomas through a cAMP-dependent way. Since the inhibition of ABCC4 seems to increase the migratory and invasive capacities of these neoplasms, the authors call attention to such a pathway as a potentially actionable therapeutic target.

Two contributions in this Special Issue deal with breast cancer [3,4], the leading cause of cancer-related death in European women. In their work, Panzetta et al. [3] study how the extracellular matrix stiffness interferes in the adhesion and migration properties of two different mammary cell lines under the exposure of two different X-ray doses. The authors conclude that the microenvironment simulating healthy tissue has a radioprotective role in preventing cell motility and invasion. Instead, a supraphysiological matrix stiffness promotes cell motility. This cellular response, called durotaxis and originally described in fibroblasts, observed in mammary cell lines reproduces the results obtained in previous experiments performed in unicellular organisms (*Dictyostelium discoideum* and *Caenorhabditis elegans*, among others) and melanoma cell lines, as it will be mentioned elsewhere in this collection [5]. On the other hand, Levine and Ogunwobi [4] focus their work on triple-negative breast cancer, a subset of around 10% of mammary carcinomas which pursues an especially aggressive clinical course. More specifically, they have centered their research on one of the six subtypes of this tumor variant, that is, the so-called claudin-low triple-negative breast carcinoma. Such spectrum of molecular variants of triple-negative breast cancer has been identified based on their specific genomic profiles. In brief, they have found that targeting the plasmacytoma variant translocation 1 (PTV1) exon 9 results in the re-expression in these tumors of the claudin 4 protein, this way inhibiting tumor cell migration. The authors stress that this finding could have important clinical implications in this specific subset of patients.

Although rare, Leydig cell tumor is probably the most frequent non-germ cell tumor in the human testis [6]. The majority of them are benign, but a small percentage (<10%) pursue a malignant course. Here, Ponikwicka-Tyszko et al. [7] have analyzed the effect of mifepristone, the selective progesterone receptor modulator, in a transgenic mouse model and two Leydig tumor cell lines. They conclude that mifepristone acts as a membrane progesterone agonist promoting Leydig cell tumor progression.

Melanoma is a classic model to analyze tumor cell migration in clinics and research [8,9] and this collection of Cell Motility and Cancer includes three contributions [10,11,12]. An Australian clinical study of 306 metastatic melanomas has found that *BRAF* + *NRAS* mutations were associated to the central nervous system and liver metastases, while *BRAF* mutation was to lymph node metastases and *NRAS* mutation with lung metastases [8], so tumor mutation status may advise to direct specifically to these sites the clinical surveillance of these patients. A recent review describes the last advances in the remodeling of melanoma cell metabolism, e.g., glycosylation and oxidative phosphorylation, along with its temporal development from nevus to metastases [9]. El-Kharbili et al. [10] delineate how keratinocytes cooperate with melanoma cells in dermal colonization through dermal-epidermal junction proteolysis induced by the Tspan8 action. Moreover, the same author has shown in other studies that its encoding gene, *TSPAN8*, acts not only in reducing matrix adherence [13] but also in promoting invasion [14]. The authors conclude that using Tspan8-blocking antibodies would prevent early melanoma from spreading [10]. Naffa et al. [11] show that PMCA4b, a plasma membrane Ca^2+^ key pump in the regulation of cytosolic Ca^2+^ concentration, regulates melanoma cell migration via remodeling the actin cytoskeleton. PMCA4b plays a key role in regulating cell polarity through F-actin rearrangement resulting in a less aggressive phenotype. Interestingly, the same group has previously shown that PMCA4b inhibits cell migration and metastatic capacities in BRAF mutant melanoma cells [15]. Kwan et al. [12] analyze the role of LRG1, a leucine-rich alpha 2 glycoprotein, in melanoma and conclude that this protein is required for metastatic dissemination but not for cell growth.

Heissig et al. [16] have contributed to this Special Issue with a review of the functional role of the epidermal growth factor-like protein-7 (EGFL7) in cancer and drug resistance. This protein is involved in cell migration and neoangiogenesis thus contributing to tumor metastases. The review includes a detailed description of the protein, its contribution to the development of a pathological tumor vessel phenotype, its role enhancing tumor immune escape, its regulation of the extracellular matrix stiffness, and its contribution to drug resistance.

The cytoskeletal dynamics involved in the epithelial–mesenchymal transition processes and their role as potential targets for cancer metastases have been reviewed by Datta et al. [17]. The key role of the cytoskeleton in cell motility is deeply analyzed in this review, from its structure and functions to its implication in epithelial–mesenchymal transition processes and its importance in multidrug resistance. The authors conclude that the interplay between cytoskeleton dynamics and epithelial–mesenchymal transition should be utilized to identify potential biomarkers.

Cheng et al. [18] review the role of peroxisome proliferator-activated receptors (PPAR) as metabolic regulators in neoplasms. PPARs are essential in reprogramming cancer-associated fibroblasts and adipocytes and regulate the paracrine and autocrine signaling of cancer-associated fibroblasts and tumor-associated macrophages/immune cells. The authors conclude that PPAR-based anticancer treatment could be achieved by modulating its physiological activity.

Readers interested in knowing how motility mechanisms of unicellular organisms can be translated to human cancer cells, and how the analysis of motility properties of cells in a wide variety of protists, worms, insects, etc., have helped to understand cell motility mechanisms in mammals, have an excellent opportunity reading the review by De la Fuente and López [5] included in this collection. The authors explain why simple organism models are necessary to understand human cell behavior. Additionally, they focus on the similarities between the locomotion system in unicellular eukaryotic organisms and human cells, the connection between external stimuli (galvanotaxis, chemotaxis, haptotaxis, barotaxis, durotaxis, etc.), migration, and cancer. They have verified that a cell migratory behavior can be modified by changes in the signals coming from the external medium (cellular associative conditioning) [19]. Likewise, the role of the nucleus in cell migration analyzed from a quantitative perspective is also a special topic in this review [5], linked to cancer, and in which the authors reflect their own previous experience [20].

Capp et al. [21] connect essential points in cancer biology such as the development of metastases with strict ecological principles. They conjecture if the so-called Parrondo’s paradox [22] may play any role in cancer biology. The paradox, defined as how combinations of losing strategies produce a winning outcome, may help to explain some particular behaviors in biological collectivities. The authors hypothesize if stability is a losing strategy for malignant cells, why should cell populations with high stochasticity be needed for long term survival and proliferation, how dormancy can be considered as a losing strategy in Parrondo’s dynamics, and if the metastatic behavior is a strategy under the paradox [21]. In the authors’ opinion, this perspective may have therapeutic implications. Alternating two losing strategies, i.e., to treat aggressively a tumor thus promoting the development of resistant clones and not to treat it at all, the cost of the investment needed to acquire drug resistance by tumor cells would need to be shared with the cost to maintain tumor cell proliferation. As proposed by Kam et al. [23], alternating a fake drug (called ersatzdroges) with a real drug may allow keeping the tumor size constant, without resistance selection. Interestingly, a close strategy promoting tumor containment has been recently proposed by Viossat and Noble [24] to avoid, or delay, tumor resistances.

Last but not least, Keller-Pinter et al. [25] review the role of syndecan-4, a transmembrane proteoglycan, in cell motility of several tumors, including melanoma, breast and lung cancers, among others, and point to this protein as a potential therapeutic target.

In conclusion, cell motility is an essential characteristic of cells, from unicellular organisms to cancer, and this Special Issue has intended to put together very different viewpoints of the intricated mechanisms involucred on it. Since cell migration is a constant event in cancer and is responsible for tumor invasion and metastases, the two dismal effects of neoplasia, this collection of articles and reviews aims to serve as a translational bridge between basic researchers and clinicians promoting interdisciplinary collaboration. We hope to meet the objective.

## References

[1] Skrzypek K., Kot M., Konieczny P., Nieszporek A., Kusienicka A., Lasota M., Bobela W., Jankowska U., Kedracka-Krok S., Kajka M. (2020). SNAIL promotes metastatic behavior of rhabdomyosarcoma by increasing EZRIN and AKT expression and regulating microRNA networks. Cancers.

[2] Kryczka J., Sochacka E., Papiewska-Pajak I., Boncela J. (2020). Implications of ABCC4-mediated cAMP efflux for CRC migration. Cancers.

[3] Panzetta V., La Verde G., Pugliese M., Artiola V., Arrichiello C., Muto P., La Commara M., Netti P.A., Fusco S. (2020). Adhesion and migration response to radiation therapy of mammary epithelial and adenocarcinoma cells interacting with different stiffness substrates. Cancers.

[4] Levine F., Ogunwobi O.O. (2021). Targeting PVT1 exon 9 re-expresses claudin 4 protein and inhibits migration by claudin-low triple negative breast cancer cells. Cancers.

[5] De la Fuente I.M., López J.I. (2020). Cell motility and cancer. Cancers.

[6] Kim I., Young R.H., Scully R.E. (1985). Leydig cell tumors of the testis. A clinicopathological analysis of 40 cases and review of the literature. Am. J. Surg. Pathol..

[7] Ponikwicka-Tyszko D., Chrusciel M., Pulawska K., Bernaczyk P., Sztacheklska M., Guo P., Li X., Toppari J., Huhtaniemi I.T., Wolczynski S. (2020). Mifepristone treatment promotes testicular Leydig cell tumor progression in transgenic mice. Cancers.

[8] Adler N.R., Wolfe R., Kelly J.W., Haydon A., McArthur G.A., McLean C.A., Mar V.J. (2017). Tumor mutation status and sites of metastasis in patients with cutaneous melanoma. Br. J. Cancer.

[9] Sobiepanek A., Paone A., Cutruzzolà F., Kobiela T. (2021). Biophysical characterization of melanoma cell phenotype markers during metastatic progression. Eur. Biophys. J..

[10] El-Kharbili M., Cario M., Béchetoille N., Pain C., Boucheix C., Degoul F., Masse I., Berthier-Vergnes O. (2020). Tspan8 drives melanoma dermal invasion by promoting proMMP-9 activation and basement membrane proteolysis in a keratinocyte-dependent manner. Cancers.

[11] Naffa R., Padányi R., Ignácz A., Hegyi Z., Jezsó B., Tóth S., Varga K., Homolya L., Hegedüs L., Schlett K. (2021). The plasma membrane Ca^2+^ pump PMCA4b regulates melanoma cell migration through remodeling of the actin cytoskeleton. Cancers.

[12] Kwan Y.P., Teo M.H.Y., Lim J.C.W., Tan M.S., Rosellinny G., Wahli W., Wang X. (2021). LRG1 promotes metastatic dissemination of melanoma through regulating EGFR/STAT3 signalling. Cancers.

[13] El-Kharbili M., Robert C., Witkowski T., Danty-Berger E., Barbollat-Boutrand L., Masse I., Gadot N., de la Fouchardière A., McDonald P.C., Dedhar S. (2017). Tetraspanin 8 is a novel regulator of ILK-driven beta1 integrin adhesion and signaling in invasive melanoma cells. Oncotarget.

[14] El-Kharbili M., Agaësse G., Barbollat-Boutrand L., Pommier R.M., de la Fouchardière A., Larue L., Caramel J., Puisieux A., Berthier-Vergnes O., Masse I. (2019). Tspan8-β-catenin positive feedback loop promotes melanoma invasion. Oncogene.

[15] Hegedũs L., Garay T., Molnár E., Varga K., Bilecz Á., Török S., Padányi R., Pászty K., Wolf M., Grusch M. (2017). The plasma membrane Ca^2+^ pump PMCA4b inhibits the migratory and metastatic activity of BRAF mutant melanoma cells. Int. J. Cancer.

[16] Heissig B., Salama Y., Takahashi S., Okumura K., Hattori K. (2021). The multifaceted roles of EGFL7 in cancer and drug resistance. Cancers.

[17] Datta A., Deng S., Gopal V., Yap K.C.H., Halim C.E., Lye M.L., Ong M.S., Tan T.Z., Sethi G., Hooi S.C. (2021). Cytoskeletal dynamics in epithelial-mesenchymal transition: Insights into therapeutic targets for cancer metastasis. Cancers.

[18] Cheng H.S., Yip Y.S., Lim E.K.Y., Wahli W., Tan N.S. (2021). PPARs and tumor microenvironment; the emerging roles of the metabolic master regulators in tumor stromal-epithelial crosstalk and carcinogenesis. Cancers.

[19] De la Fuente I.M., Bringas C., Malaina I., Fedetz M., Carrasco-Pujante J., Morales M., Knafo S., Martinez L., Pérez-Samartin A., López J.I. (2019). Evidence of conditioned behavior in amoebae. Nat. Commun..

[20] De la Fuente I.M., Bringas C., Malaina I., Regner B., Pérez-Samartín A., Boyano M.D., Fedetz M., López J.I., Pérez-Yarza G., Cortés J.M. (2019). The nucleus does not significantly affect the migratory trajectories of amoeba in two-dimensional environments. Sci. Rep..

[21] Capp J.P., Nedelcu A.M., Dujon A.M., Roche B., Catania F., Ujvari B., Alix-Panabières C., Thomas F. (2021). Does cancer biology rely on Parrondo’s principles?. Cancers.

[22] Cheong K.H., Koh J.M., Jones M.C. (2019). Paradoxical survival: Examining the Parrondo effect across biology. BioEssays.

[23] Kam Y., Das T., Tian H., Foroutan P., Ruiz E., Martinez G., Minton S., Gillies R.J., Gatenby R.A. (2015). Sweat but not gain: Inhibiting proliferation to multidrug resistant cancer cells with “ersatzdroges”. Int. J. Cancer.

[24] Viossat Y., Noble R. (2021). A theoretical analysis of tumor containment. Nat. Ecol. Evol..

[25] Keller-Pinter A., Gyulai-Nagy S., Becsky D., Dux L., Rovo L. (2021). Syndecan-4 in tumor cell motility. Cancers.

